# Research and Remedies

**Published:** 1912-10-19

**Authors:** 


### RESEARCH AND REMEDIES.

### The Treatment of Amœbic Dysentery.

The ipecacuanha treatment of dysentery has long been a favourite with specialists in tropical medicine; Sir Patrick Manson has always advocated it strenuously. Now Professor Leonard Rogers proposes to modify it by using emetine, one of the active principles, instead of the crude drug. He has shown that the Amœba dysenteriæ is rapidly destroyed in the stools of dysenteric patients by even one part in a hundred thousand of emetine. Consequently he has been administering doses of $\frac{1}{6}$ grain of emetine hydrochloride hypodermically to patients with great success. He has even given doses of $\frac{1}{2}$ grain—equivalent to 45 grains of ipecacuanha—without upsetting the patients' stomachs. Curiously, too, these large doses of emetine were tolerated by patients who had previously experienced nausea and vomiting under the usual ipecacuanha treatment.

### The Organism of Syphilis.

After many observers had spent years in efforts to discover the micro-organism of syphilis, Schaudinn and Hoffman in 1905 described a spirochæta which has ever since been accepted as the genuine infecting parasite of this disease. The experience of the ensuing seven years since this discovery has rendered the position of this organism more and more secure in the minds of bacteriologists and syphilologists. Mr. J. E. R. McDonagh, however, is not satisfied that the whole truth about the Spirochæta pallida has yet been discovered, and he propounds in the *Lancet* some novel and very ingenious researches into the life-history of this parasite. He believes that the spirochæta is only one stage in the development of the syphilitic organism: indeed, he regards it as simply the male gamete in the reproductive cycle of the parasite. The female gamete is a spherical body; and, although he has not witnessed the conjugation of these forms, he gives a description of what he believes to be the zygote which they form. This cell divides into four sporoblasts, which in turn form sporozoites; and the latter penetrate the mononuclear leucocytes, after which the sexual cycle starts again. This highly interesting theory has been built up upon observations upon the minute structure of syphilitic lymphatic glands, chancres, and other tissues. It may quite likely stand the test of further investigation; but Mr. McDonagh has indulged in a good deal of deductive reasoning from preconceived hypotheses in his search for non-spiral forms of the parasite. If his account of the life-history of the syphilitic organism is correct, clearly it ought no longer to be called by its present name. He suggests instead *Leucocytozoon syphilis*.

